# A Case Report of Dilated Biventricular Heart Failure from Hyperthyroidism: A Rare Presentation

**DOI:** 10.7759/cureus.2410

**Published:** 2018-04-02

**Authors:** Rizwan Ali, Arooj Tahir, Kanna V Posina

**Affiliations:** 1 Internal Medicine, Rapides Regional Hospital, Alexandria, La; 2 Cardiology, Rapides Regional Hospital, Alexandria, La

**Keywords:** hyperthyroidism, biventricular heart failure

## Abstract

Hyperthyroidism is a common metabolic disorder with many cardiovascular manifestations. In rare cases, untreated hyperthyroidism can lead to thyrotoxic cardiomyopathy with severe left ventricular (LV) dysfunction. This case report aims to discuss the pathogenesis of heart failure in hyperthyroidism and the available treatment options.

A 51-year-old male with a past history of untreated hyperthyroidism presented to our hospital for the evaluation of shortness of breath and dysphagia. Workup revealed atrial flutter and severe biventricular dilated cardiomyopathy. Stabilization thyroidectomy was performed due to dysphagia, and treatment with oral antithyroid medications was initiated. The patient was discharged on synthroid and beta-blockers.

Untreated hyperthyroidism can lead to biventricular failure even in the young. Untreated hyperthyroidism leads to significant mortality and morbidity. Untreated hyperthyroidism is associated with atrial fibrillation, heart failure, pulmonary hypertension (PH), and angina-like symptoms. Further studies should be done to evaluate the pathogenesis of Graves/Goiter hyperthyroidism and the least-invasive, safe, and definitive treatment options should be discovered. Current treatment options are limited and include medication that needs to be taken lifelong; they are associated with toxicity. Radioactive iodine ablation comes with the drawback of long-term replacement therapy. The last option is surgery, which is invasive and has its own complications.

## Introduction

Hyperthyroidism is a common metabolic disorder with cardiovascular manifestations. It often causes classical high-output heart diseases because of decreased systemic vascular resistance and increased resting heart rate, left ventricular (LV) contractility, blood volume, and cardiac output [1-2]. However, thyrotoxic cardiomyopathy with severe LV dysfunction is rare. Heart failure (HF) is most commonly seen as a result of longstanding, often untreated, thyrotoxicosis with coexistent atrial fibrillation (AF). HF is a major cause of morbidity and mortality in Europe and in the United States and is responsible for a high rate of hospitalization [3-4]. Despite the advancement in HF treatment in the past 15 years, the prognosis of this dysfunction remains poor [5]. Thyroid dysfunction is a modifiable risk factor for patients who are at risk of HF [6-7].

## Case presentation

A 51-year-old male with a past medical history of hypertension and hyperthyroidism presented to the emergency department with symptoms of cough, shortness of breath, palpitation, dysphagia, pedal edema, and subjective fever. He was not taking medication for hyperthyroidism.

**Figure 1:**
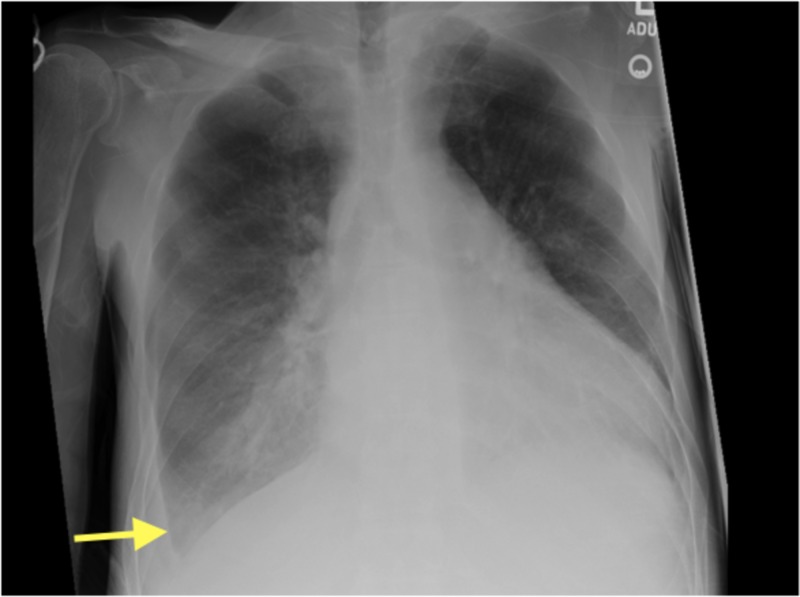
Chest X-Ray Arrow showing right pleural effusion

The exam showed tachycardia, enlarged thyroid, crackles on lung auscultation, and edema feet. Further chest X-ray (CXR) showed pneumonia with right pleural effusion, as shown in Figure 1.

**Figure 2:**
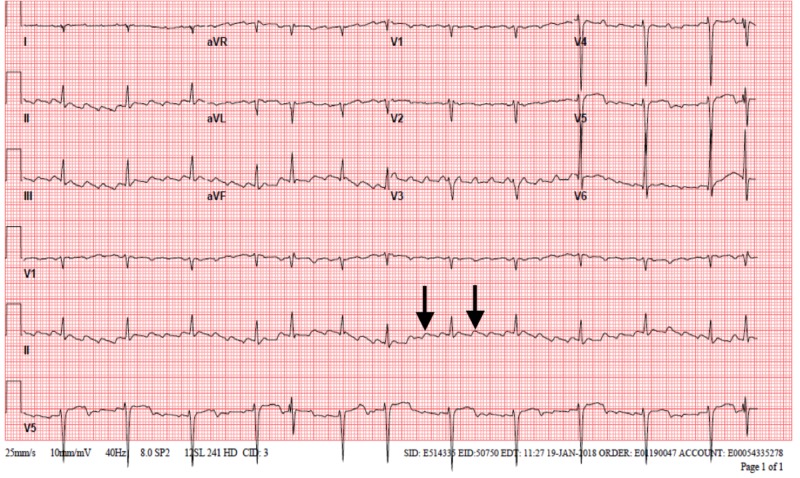
EKG showing atrial flutter Arrows showing sawtooth pattern EKG: electrocardiogram

Electrocardiography (EKG) showed atrial flutter with a variable atrioventricular block, as shown in Figure 2.

Other significant abnormal labs were as shown in Table 1.

**Table 1:** Thyroid function test

TSH	Free T4	Free T3
<0.005	>7.5	25.1

The patient was started on levaquin for pneumonia. For atrial flutter, an echocardiogram was ordered and cardiology was consulted. The patient was started on propylthiouracil (PTU) 50 mg three times a day and a beta-blocker.

**Figure 3:**
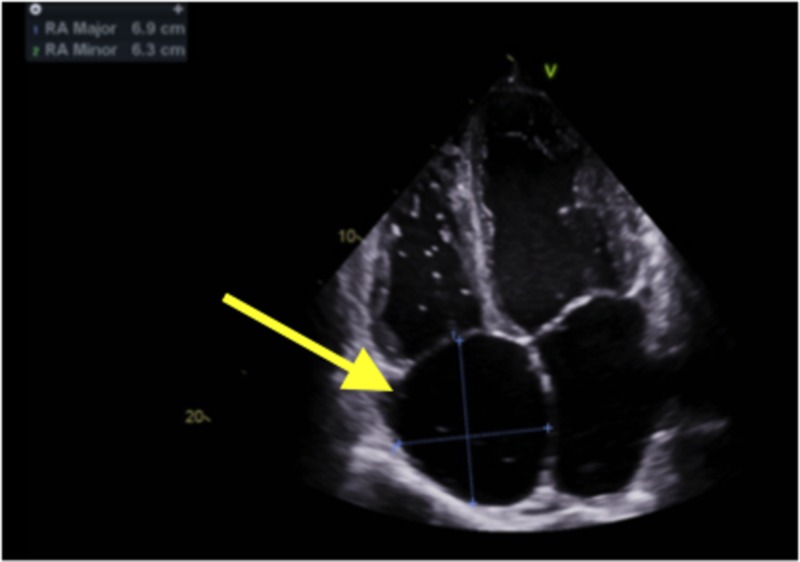
Echocardiogram: Arrow pointing toward dilated right atrium

**Figure 4:**
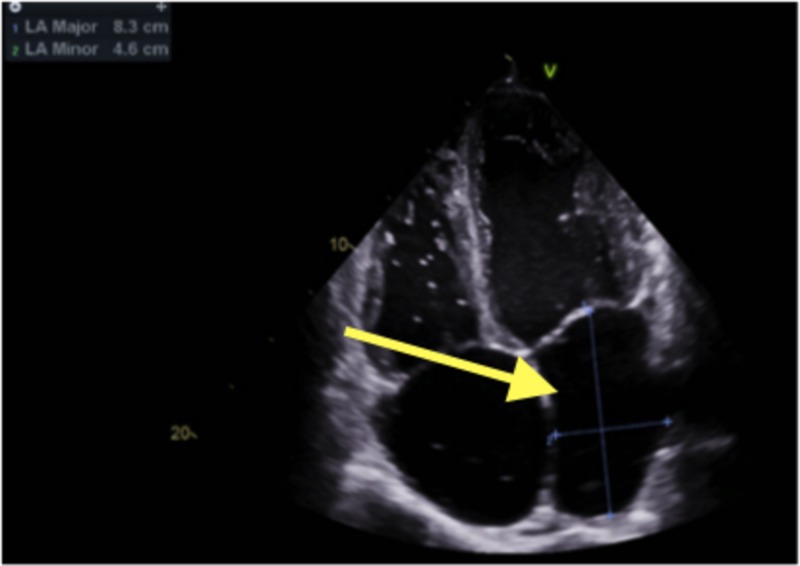
Echocardiogram: Arrow pointing toward dilated left atrium

**Figure 5:**
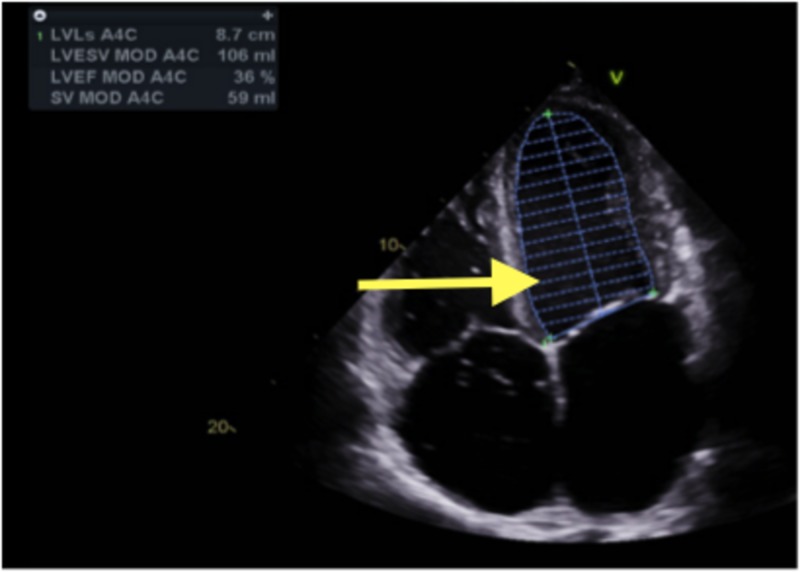
Echocardiogram: Arrow pointing toward dilated left ventricle

The echocardiogram showed mild biventricular dilatation. The left ventricular systolic function was markedly reduced with an ejection fraction of 25% to 30% with severe diffuse hypokinesis. The right ventricular systolic function was moderately reduced, with a markedly elevated right ventricular systolic pressure of 59 mmHg. There was also marked biatrial enlargement. Echocardiogram findings are shown in Figures 3-5.

**Figure 6:**
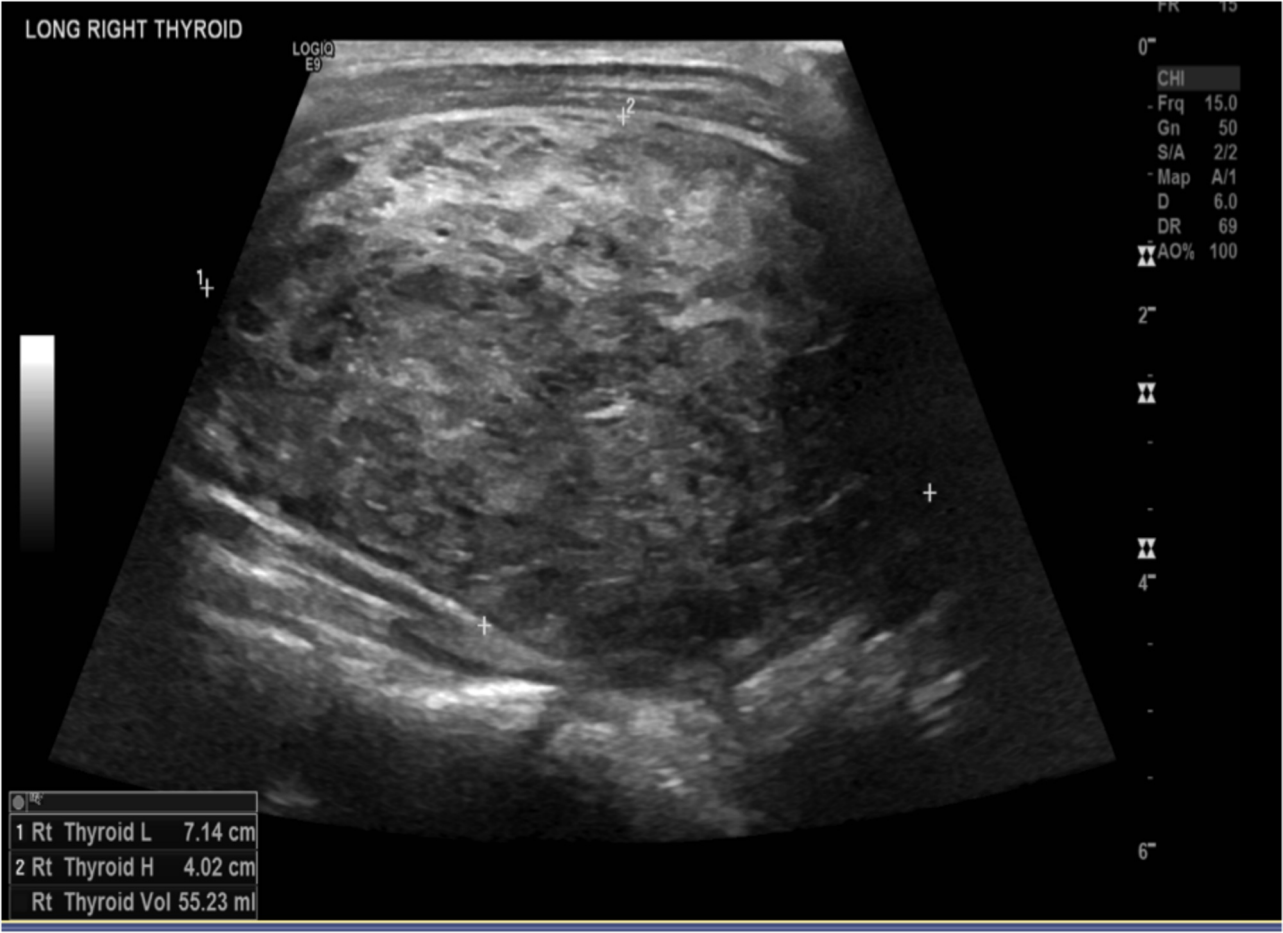
Right thyroid lobe with dimensions

**Figure 7:**
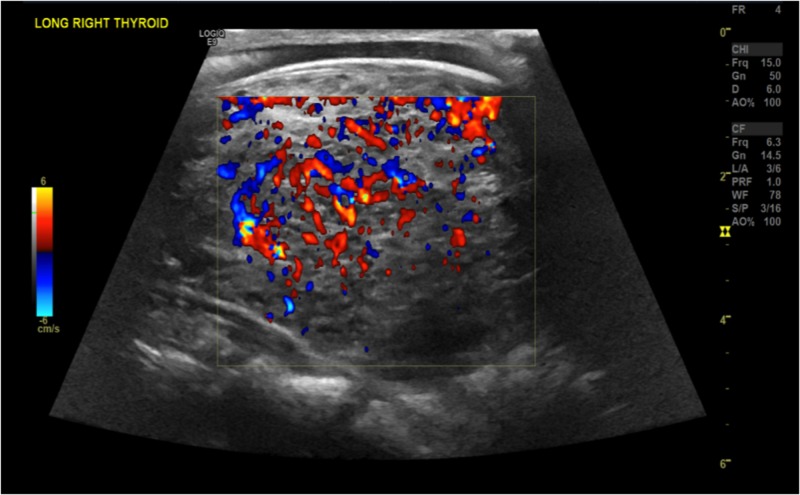
Doppler showing vascularity in the right thyroid lobe

**Figure 8:**
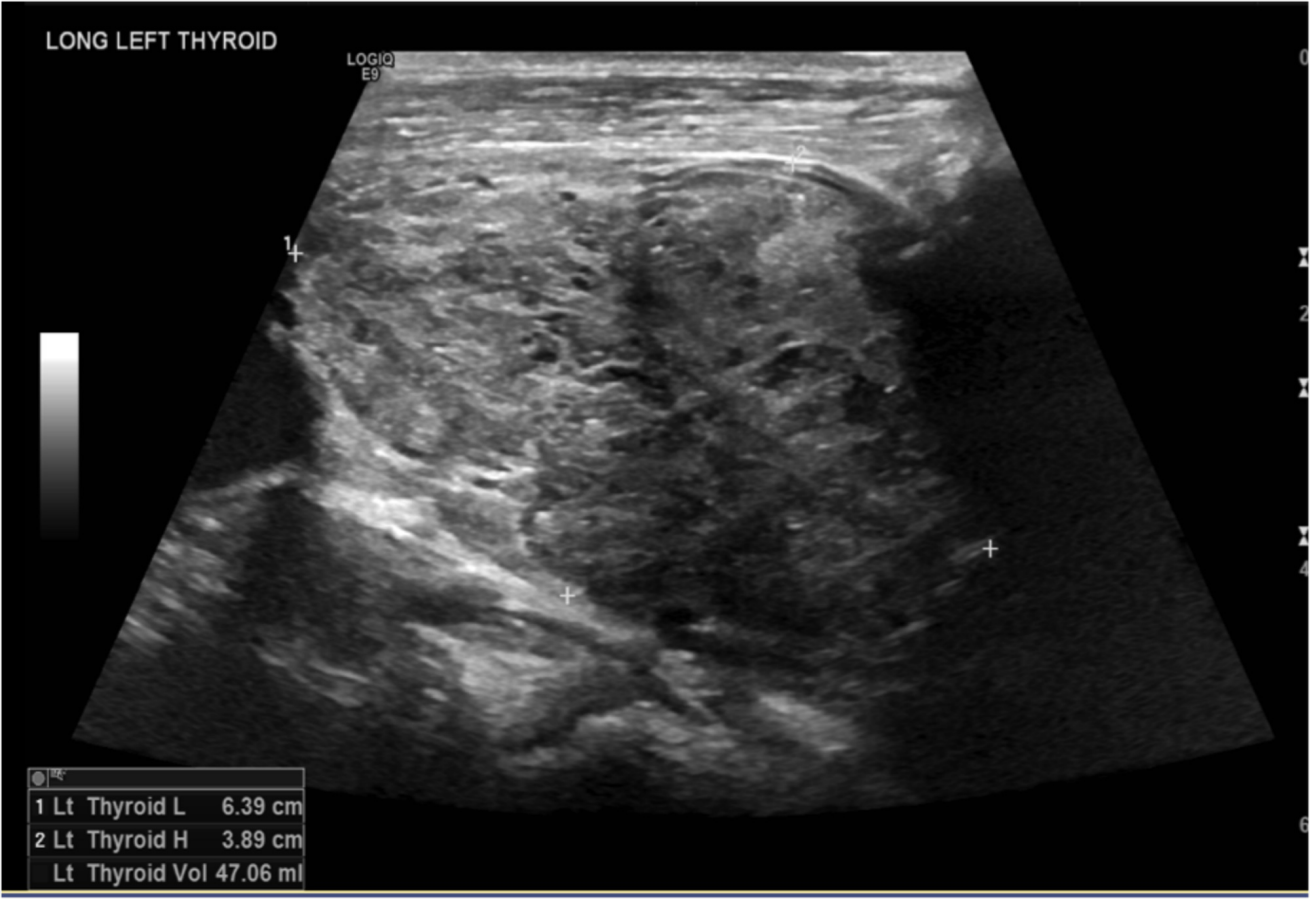
Left thyroid lobe with dimensions

**Figure 9:**
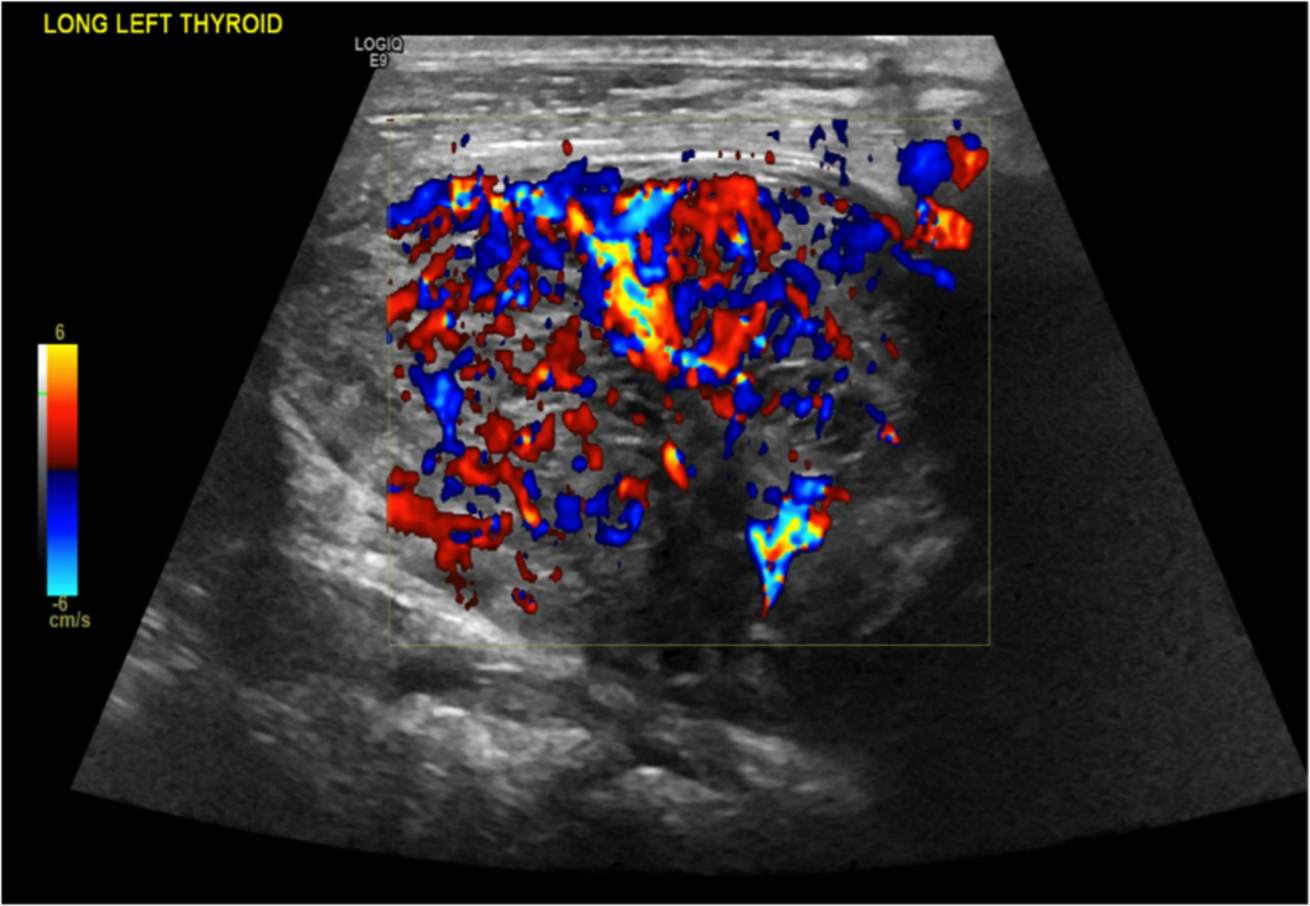
Doppler showing vascularity in the left thyroid lobe

**Figure 10:**
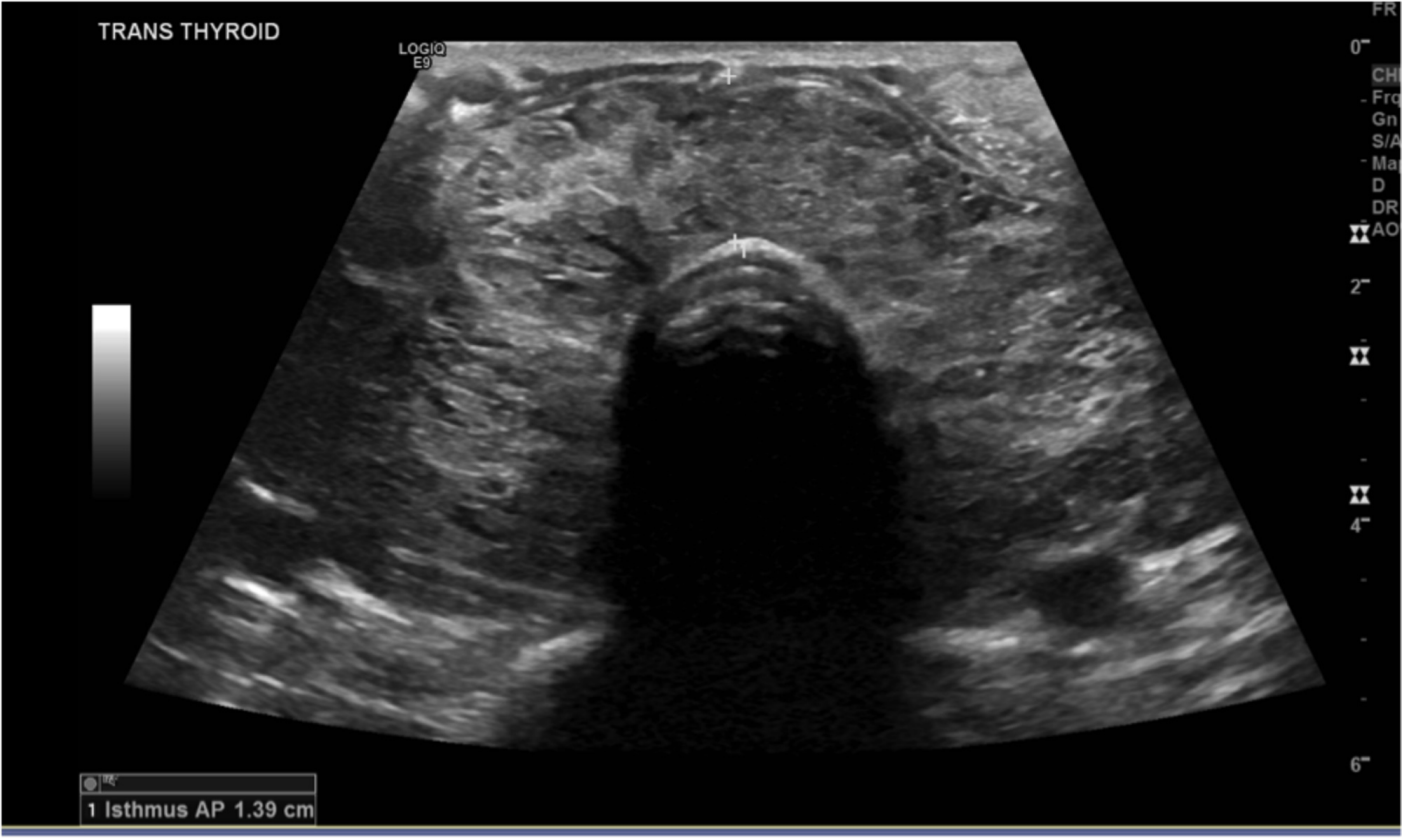
Isthmus of thyroid

**Figure 11:**
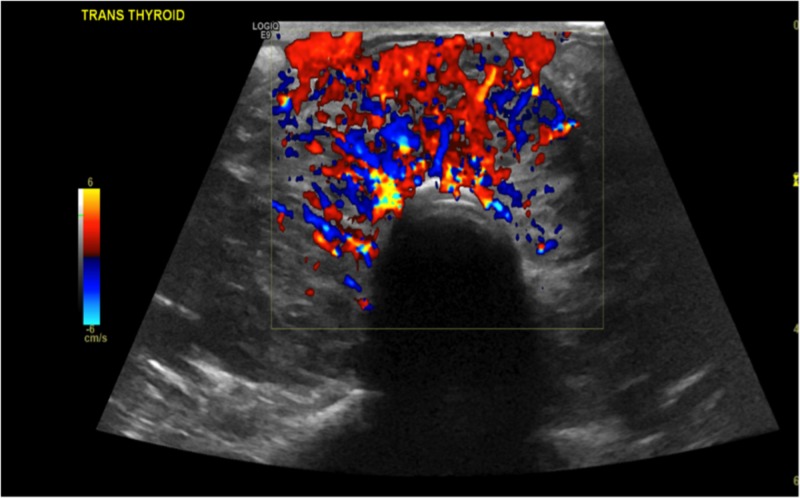
Doppler showing vascularity in the isthmus of thyroid

The patient was started on the beta-blocker and anticoagulation. A stress test was done that showed a small, fixed defect in the apex, but no evidence of ischemia. The patient was also diuresed with diuretics due to the volume overload. A thyroid ultrasound was ordered that showed that the right and left lobes were enlarged and hyperemic, measuring 7.1 X 4.0 X 4.0 and 6.4 X 3.9 X 4.0, cm, respectively. The thyroid ultrasound findings are shown in Figures 6-11.

Findings were consistent with goiter. General surgery recommended thyroidectomy due to the mass effect of goiter. A thyroidectomy was performed without complications. The pathology showed multinodular goiter and the patient had an uneventful rest in the hospital. He was discharged on a beta-blocker, synthroid, lasix, and rivaroxaban.

## Discussion

This patient presented with severe but reversible systolic LV dysfunction due to hyperthyroidism. He was relatively young, which indicates that the development of overt congestive heart failure (CHF) due to hyperthyroidism is not limited to the elderly population, but can develop in younger patients if hyperthyroidism is left untreated for a long period of time.

Hyperthyroidism, usually due to Graves’ disease, is fairly commonly encountered in clinical practice and can present with a wide variety of signs and symptoms. Typically, it presents with the features of heat intolerance, weight loss, sweating, palpitation, tremors, and hyper defecation. If left untreated, it can cause heart failure. Occasionally, it presents with heart failure in the absence of any classic symptoms of hyperthyroidism, as is the case with the apathetic hyperthyroidism seen in the elderly [8].

The cardiac effects of the thyroid hormone have been known for more than a century.

Thyroid hormone exerts its cardiac effects indirectly through its effect on the vasculature and body metabolism and directly through its effect on the heart. Peripherally, tri-iodothyronine (T3) has been shown to decrease systemic vascular resistance (SVR) by promoting vaso-dilatation [9]. This action is mediated by the direct effect of T3 on vascular smooth muscle [10]. The resulting decrease in SVR activates the renin-angiotensin-aldosterone system, leading to retention of sodium (Na+) and fluid. Thyroid hormone also increases erythropoiesis. The net effect is a resultant increase in the total blood volume and stroke volume. At the myocyte level, T3 enters the cell via specific transport proteins, resulting in the enhanced contractility and relaxation of the myocardial cells through transcription and non-transcription-mediated effects. The transcriptional effects lead to increased contractility through effects on the release and uptake of sarcoplasmic reticular calcium (Ca++) and the phosphorylation of phospholamban. The nontranscriptional effects are mediated by the effect of thyroid hormone on various ion channels. These cardiac effects, coupled with a generalized increase in tissue metabolism, low SVR, and an increase in total blood volume, lead to a high cardiac output state in hyperthyroidism.

Clinically, thyroid hormone can have a wide variety of effects on the heart, ranging from sinus tachycardia and AF, to dilated CHF.

Clinically significant CHF due to hyperthyroidism is considered a rare occurrence. Initially, in the course of the disease, the patient is in a high cardiac output state, due to the factors mentioned above, limiting only exercise tolerance. Later in the course of the disease, if untreated, the patient can develop severe systolic dysfunction with overt signs and symptoms of heart failure. This is more commonly seen in patients with a pre-existing heart disease, such as ischemic, hypertensive, or alcoholic cardiomyopathy, the former being more common in the elderly. Although the exact etiology of CHF in hyperthyroidism is unclear, the concept of “tachycardia-induced cardiomyopathy” secondary to prolonged sinus tachycardia or AF with a rapid ventricular response is more plausible, as LV dysfunction commonly improves with adequate control of the heart rate long before the euthyroid state is restored.

The treatment of CHF should be aimed at correcting hyperthyroidism with oral antithyroid medication. The first line of treatment of CHF secondary to hyperthyroidism is a beta blocker, except in patients with marked hypotension, reversible airway disease, and marked bradycardia, especially with a second- or third-degree atrioventricular block. Beta-blockers not only help ameliorate the noncardiac symptoms of the disease but also decrease the heart rate by controlling sinus tachycardia or decreasing the ventricular response to AF by action on the β1 receptors in addition to other unproven actions.

## Conclusions

Hyperthyroidism, if untreated, can lead to overt dilated HF and death. Hyperthyroidism treatment should be taken seriously. Definitive treatment is radioactive iodine gland ablation vs. surgery. Initially, the patient should be stabilized with oral antithyroid medication. The treatment of hyperthyroidism has not changed greatly in the past several decades. Future research should be directed toward better understanding the pathogenesis of Graves/goiter hyperthyroidism to direct therapy at the underlying cause of the hyperthyroidism and to obtain a cure that is safe, conservative, and definitive. Current treatment options are limited and include medication that needs to be taken lifelong; this is associated with their toxicity. Radioactive iodine ablation comes with the drawback of long-term replacement therapy. The last option is surgery, which is invasive and has complications.

## References

[1] Klein I, Danzi S (2007). Thyroid disease and the heart. Circulation.

[2] Choudhury RP, MacDermot J (1988). Heart failure in thyrotoxicosis, an approach to management. Br J Clin Pharmacol.

[3] Setoguchi S, Stevenson LW (2009). Hospitalizations in patients with heart failure: who and why. J Am Coll Cardiol.

[4] Lee DS, Austin PC, Rouleau JL, Liu PP, Naimark D, Tu JV (2003). Predicting mortality among patients hospitalized for heart failure: derivation and validation of a clinical model. JAMA.

[5] Roger VL, Weston SA, Redfield MM, Hellermann-Homan JP, Killian J, Yawn BP, Jacobsen SJ (2004). Trends in heart failure incidence and survival in a community-based population. JAMA.

[6] Gerdes AM, Lervasi G (2010). Thyroid replacement therapy and heart failure. Circulation.

[7] Biondi B, Kahaly GJ (2010). Cardiovascular involvement inpatients with different causes of hyperthyroidism. Nat Rev Endocrinol.

[8] Williams GH, Braunwald E (1992). Endocrine and Nutritional Disorders and Heart Disease.

[9] Park KW, Dai HB, Ojamaa K, Lowenstein E, Klein I, Sellke FW (1997). The direct vasomotor effect of thyroid hormone on rat skeletal muscle resistance arteries. Anesth Analg.

[10] Ojamaa K, Klemperer JD, Klein I (1996). Acute effects of thyroid hormone on vascular smooth muscle. Thyroid.

